# The economics of psychedelic-assisted therapies: A research agenda

**DOI:** 10.3389/fpsyt.2022.1025726

**Published:** 2022-12-05

**Authors:** Elliot Marseille, Stefano Bertozzi, James G. Kahn

**Affiliations:** ^1^Global Initiative for Psychedelic Science Economics (GIPSE), University of California, Berkeley, Berkeley, CA, United States; ^2^Center for Global Health Delivery Diplomacy and Economics, University of California, San Francisco, San Francisco, CA, United States; ^3^School of Public Health, University of California, Berkeley, Berkeley, CA, United States; ^4^School of Medicine, Philip R. Lee Institute for Health Policy Studies, University of California, San Francisco, San Francisco, CA, United States

**Keywords:** psychedelics, health economics, cost-effectiveness, psychiatry, MDMA, psilocybin

## Abstract

After a long hiatus, psychiatry is undergoing a resurgence of interest in psychedelic drugs as therapy for a wide range of mental health disorders Accumulating clinical evidence suggests substantial potential for psychedelics used in a therapeutic context, as treatment for, among other disorders, depression, post-traumatic stress disorder (PTSD), and addictions to tobacco, opioids and alcohol. As soon as 2024, powerful new therapeutic modalities could become available for individuals with mental health problems refractory to traditional therapies. Yet research has lagged on economic considerations, such as costs and cost-effectiveness, the economic effects of widespread implementation, pricing, and economic appraisal's methodological considerations relevant to psychedelic therapies. These issues are critical if psychedelic therapies are to become widely accessible. We describe six types of economic analyses and their rationale for decisions and planning including the needs of health care payers. We also outline desirable features of this research, including scientific rigor, long horizons, equity, and a global view.

## Background

Mental health disorders are the fifth leading cause of Disability-Adjusted Life Years (DALYs), (1), affecting 10.7% of the global population in 2017 (2). Depression represents about a third of this burden, as do anxiety disorders and post-traumatic stress disorder (PTSD), followed by alcohol and drug use disorders at 13.1 and 8.4%, respectively. The remaining 10% consists of bipolar disorder, schizophrenia, and eating disorders (2). In the United States 21.0% of adults live with mental illness, including 5.6% with serious mental health conditions (3).

Current therapies help a significant portion of people with mental health disorders. Nevertheless, many patients do not respond adequately (4) or cannot tolerate the side effects of interventions such as, for depression, selective serotonin reuptake inhibitors (SSRIs) and electroconvulsive therapy (5–10). Psychotherapeutic approaches also fail to help a substantial portion of depressed patients (9, 11, 12). Approximately 50% of PTSD patients do not meaningfully respond to current pharmacological and psychotherapeutic treatments (13–15). A 2000 review of drug dependence and its treatments found that 40–60% of patients treated for alcohol and other substance use disorders reverted to active use within a year following treatment (16). The need for more effective mental health treatments is widely acknowledged (17).

In this context, many clinicians and the public are encouraged by recent favorable clinical reports for novel therapies incorporating psychedelic drugs to treat anxiety and depression including treatment-resistant depression and end-of-life distress (18–26); PTSD (21, 23, 27–29). Preliminary data also suggest potential benefits for addictions such as tobacco (30), opioid (31), and alcohol use disorder (32, 33), as well as eating disorders, social anxiety, cluster headaches, OCD and ADHD (34–36). Some of this evidence indicates that new psychedelic-assisted therapies may be effective not only in managing serious psychiatric conditions, but often in inducing long term remission. Despite these generally encouraging findings, no psychedelic-assisted therapy has yet been adopted into national guidelines; see for example the Canadian Network for Mood and Anxiety Treatments Task Force recommendations (37). Nor have any previously illegal psychedelic drugs been approved by a relevant regulatory agency as a legal medicine. Continued research by non-profits and, increasingly, the private sector, is focused on the safety and efficacy of the new therapies.

The economic implications of the metal health burden are huge. In the U.S., the societal economic burden of PTSD in 2018 was $232 billion (38), and of major depression was $210.5 billion in 2010 (39). Yet little investigation has been conducted on the economics of the new therapies. What do they cost per person and for society? What are the potential savings from averted illness? What are the other economic benefits? What are the net costs and cost-effectiveness, for health care payers and society? Yet these questions must be addressed if new therapies with proven clinical benefit are to be embraced by insurers and thus to become accessible at scale.

In this article, we review the economic evaluation agenda for psychedelic therapies, preceded by a brief review of clinical evidence.

The precise definition of “psychedelic” (from the Greek roots meaning “Mind-manifesting”) is somewhat controversial. For our purposes, psychedelic drugs include the “classic” serotonergic hallucinogenic agents such as lysergic acid diethylamide (LSD), psilocybin and 5-methoxy-N,N-dimethyltryptamine (DMT), and compounds such as ibogaine, 3,4-Methylenedioxy methamphetamine (MDMA) and its analogs, and ketamine all of which are profoundly mind-manifesting but have different mechanisms of action from the “classic” psychedelics.

A primer on methods for heath economics evaluation is beyond the scope of this article, though a number of excellent books and articles are available to interested readers (40–42).

## Selective overview of the clinical research

A full description of completed and ongoing clinical research on psychedelic-assisted therapy is described elsewhere (34, 43). We have selected three focus areas where: (1) clinical and economic research is relatively advanced (MDMA-assisted therapy for PTSD); (2) there is the potential to affect a disorder of particularly large public health importance (psilocybin for major depressive disorder); and (3) psychedelic therapy can affect a major non-psychiatric public health issue (psilocybin for tobacco cessation). All psychedelic interventions include major counseling components.

### MDMA to treat PTSD

In May 2021, the first of two phase 3 trials was reported: 67% receiving MDMA no longer met diagnostic criteria for PTSD, vs. 32% with placebo (28). The Food and Drug Administration (FDA) may approve MDMA by 2024.

### Psilocybin for depression

The first trial, with an open-label design, found a large benefit at 6 months (44). In 2020, a wait-list controlled randomized trial found that 71% of participants showed clinically significant response at week 4 (45). A double-blind randomized controlled trial (RCT) published in 2021 comparing psilocybin with escitalopram, a selective serotonin-reuptake inhibitor (SSRI), for patients with chronic major depression found no significant difference in depression scores, though psilocybin was superior on secondary measures of depression and well-being (18).

### Psilocybin for tobacco addiction

An open-label pilot study had promising findings (30). Preliminary results from 25 subjects in a phase 2 trial found that at 12-months, 47% of the psilocybin group had biologically-confirmed abstinence compared with 20% with placebo (46). In October, 2021, the National Institutes of Health (NIH) awarded $4 million to Johns Hopkins to support expanded research into psilocybin to treat smoking, representing the first grant to support psychedelic therapies research in over 50 years (47, 48).

## The agenda for economic analyses of psychedelic-assisted therapy

To date, three peer-reviewed articles have been published (by us) on the economics of psychedelic therapies, all on MDMA for PTSD. The first, a cost-effectiveness analysis based on the pooled results of phase 2 trials, showed that MDMA-AT was likely to generate net savings to health payers by reducing overall health care costs. The second updated this analysis with the more favorable phase 3 trial results and found correspondingly more favorable economics. The third explored the health benefits and medical cost savings to the U.S. for different scale-up rates (49).

It is unsurprising that economic analyses lag behind clinical research. Until a novel intervention demonstrates safety and efficacy, and thus the possibility of becoming FDA-approved, there is little reason to devote major resources to economic analyses. However, in view of rapid clinical research progress, economics seems more urgent. The anticipated access to decriminalized psychedelics in Oregon and elsewhere adds to this impetus.

**Figure 1:**
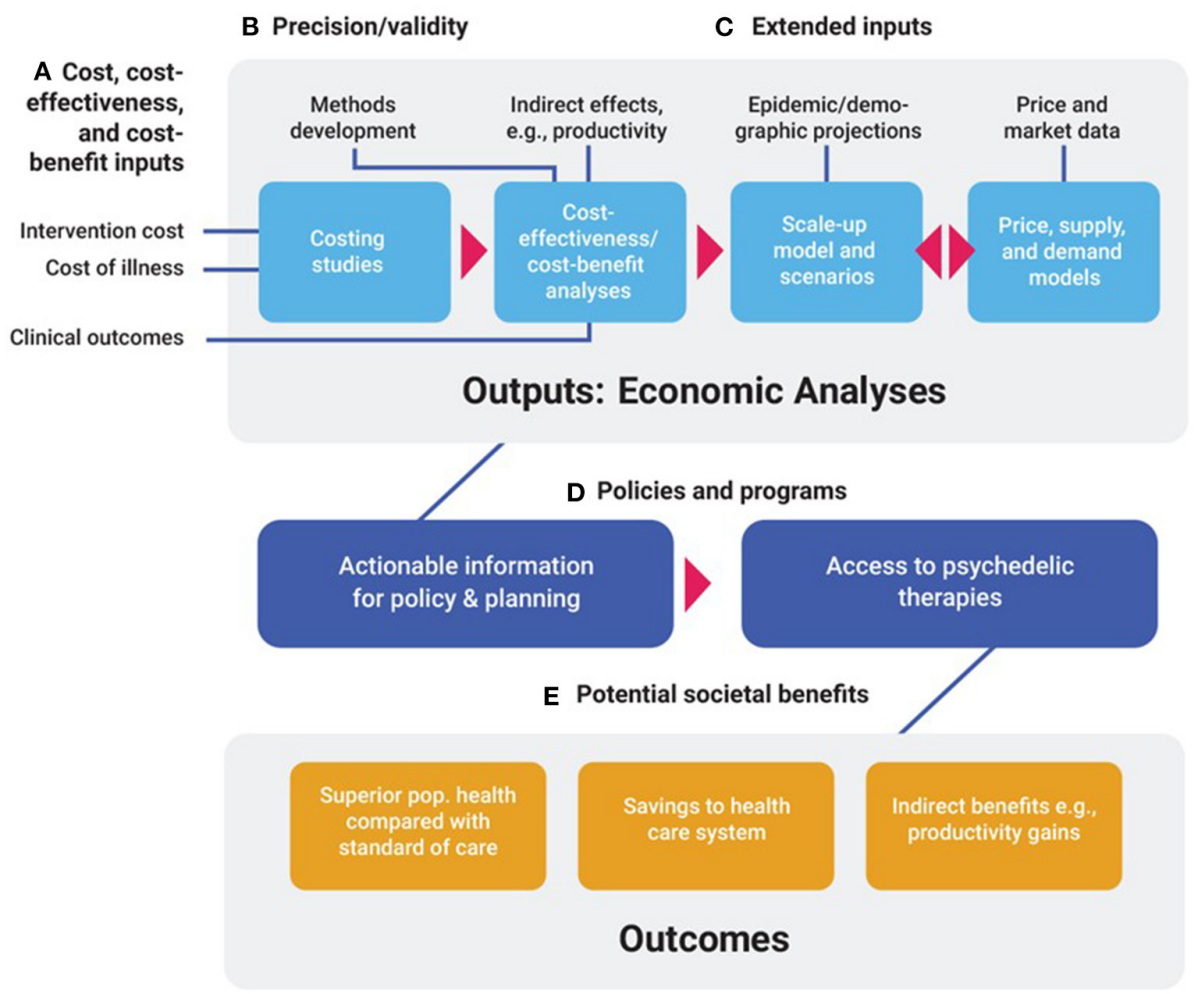
Economic evaluations of psychedelic-assisted therapies: Inputs, outputs, outcomes. **(A)** Intervention costs, health care costs (Costs of illness), and clinical outcomes are broad classes of inputs needed for costing studies, and cost-effectiveness and cost-benefit analyses. **(B)** Precision and validity of economic models are enhanced by the development of methods that accurately portray costs and benefits of psychedelic therapies. **(C)** Extended inputs such as indirect economic effects; epidemic and demographic projections; and price and market data are required for cost-effectiveness analyses from a societal perspective, scale-up, and price models, respectively. These four major types of economic analytic outputs can then inform. **(D)** Policies and programs designed to deliver access to psychedelic therapies of demonstrated cost-effectiveness. Access to these therapies generate. **(E)** Societal benefits such as enhanced health outcomes, savings to the health care system, and indirect benefits such as productivity gains.

We anticipate six distinct areas of economics research that will be useful in shaping policy and programs. These include costing, cost-effectiveness and cost-benefit analyses, scale-up and impact analyses, market and price evaluations, and methods development. Each is described below and in Table 1, and how they relate is shown in Figure 1.

**Table 1:** Six types of health economic research and their application to the assessment of psychedelic therapies.

**Type of economic research**	**Application**	**Examples for psychedelic therapy**
Costing	Characterize resources and costs to deliver intervention and to provide care for persons with relevant disorder.	Cost of delivering MDMA for PTSD adjusted for potential savings in future medical care.
Cost-effectiveness analysis	Estimate “health value for money”: Divide net costs by health gains, measured in natural units (e.g., depression cases in remission) or composite measures such as Quality-Adjusted Life-Years (QALYs).	1. Psilocybin for major depression compared with standard of care. 2. Care delivery models, e.g., individual vs. group; two vs. one clinician.
Cost-benefit analysis	Compare the cost of intervening with the financial value of benefits obtained.	Psilocybin for smoking cessation compared with standard of care: Ratio of dollar valuation of health benefits divided by intervention costs.
Scale-up and budget analysis	Estimate the system-wide costs and health benefits of large-scale use, for plausible rates of implementation.	Psilocybin for alcohol use disorder: Aggregate net costs and QALYs gained of implementation at scale.
Price analysis	Derive prices for an intervention that maximize an objective such as profit, revenue, or access / social benefit.	Establish appropriate price per unit of MDMA, psilocybin or other psychedelic medicine.
Methods development	Identify novel approaches to portrayal of health effects of psychedelic therapies.	1. Incorporate positive health states into economic analyses. 2. Modeling positive and negative health and economic impacts of non-clinical psychedelics use.

***Cost analysis*** is a linchpin of economic assessment. Costing studies are done by quantifying resources needed (e.g., hours of counselor time) and their unit prices. They answer questions such as, “What does it cost to deliver the psychedelic intervention? What are the general costs of treating the disease? How do overall medical costs change with successful treatment?”

***Cost-effectiveness analysis*** (CEA) is the most frequently-used tool to assess health program or policy choice when considering both cost and health benefits. Health benefits are typically denominated in Quality-Adjusted Life-Years^1^ (QALYs), or in “natural” metrics such as deaths or cases of disease averted. Results are expressed as incremental cost-effectiveness ratios, eg cost per QALY gained (50). As with any important new health technology, psychedelic therapies will need credible cost-effectiveness analyses if they are to become mainstream (51–53). Particularly in view of residual stigma from the war on drugs, insurers are unlikely to approve routine use of these treatments without credible estimates of delivery cost, potential downstream medical savings, and associated health benefits. These types of estimates are also needed if psychedelic therapies are to be approved in Europe. The European Medicines Agency collaborates with the Health Technology Assessment (HTA) bodies in respective EU countries. These HTAs, in turn, assess the relative effectiveness and cost-effectiveness of new medicines and their impact on healthcare budgets (54, 55).

CEAs of psychedelic therapies should be conducted from the perspective of insurers, as these are the gatekeepers of access. For example, insurance companies are subject to high rates of patient turnover (56). This means that for many patients, the relatively high up-front cost of psychedelic therapies will not be re-couped by the payer since patients will have exited the plan before those costs are fully recovered in the form of reduced medical care spending. Thus, for gaining a realistic estimate of the effect on cash flow and budgets over time, CEAs, when combined with company-specific knowledge of turnover rates, are indispensable.

But important societal benefits are not captured by those who pay for care, creating a tendency to under-invest in the new therapies. For example, health insurers may be concerned that they bear substantial up-front costs of psychedelic-assisted therapy whereas reductions in health care utilization accrue only over years, after many patients have migrated to other insurers. Other misaligned incentives concern increased productivity by people returning to employment; and reduced absenteeism and “presenteeism” among employed individuals. Still other societal benefits fall outside of health care and employment, such as potential reductions in domestic violence (57), incidence of “driving under the influence,” and involvement with the criminal justice system (58, 59). In keeping with recommendations of the Second Panel on Cost-Effectiveness in Health and Medicine (42), the psychedelic therapy economic research agenda should quantify such broader societal benefits. Such analyses can build political acceptance for the clinical use of these formerly illegal and stigmatized drugs.

CEAs can also examine delivery options. For example, our recent CEA of the phase 3 MDMA trial assessed the cost-effectiveness of a regimen with three active MDMA sessions for the treatment of PTSD compared with the two sessions employed in the Phase 2 trials (60). Other examples pertain to the relative costs and benefits of individual vs. group sessions and clinical prioritization: all patients with major depressive disorder (MDD), vs. only those with treatment-resistant MDD.

Assessments of severe adverse events (SAEs) need to be included in cost-effectiveness analyses and other economic appraisals. For example, 12 patients in Compass Pathway's Phase IIb trial (n = 233) of their patented psilocybin formulation to treat treatment-resistant depression experienced one or more SAEs including such issues as suicidal behavior and intentional self-injury [COMPASS (61)]. Both the additional medical care cost, such as ambulance and emergency medical services, and the loss of health and well-being (typically measured in QALYs) due to SAEs need to be included in the overall economic assessment. Because patients with severe depression or other disorders may suffer elevated rates of such events compared with the general population in the absence of psychedelic therapy, it will be important to isolate the treatment-attributable portion of the reported incidence of SAEs.

Finally, cost-effectiveness analyses of psychedelic therapies to date have been performed for well-resourced and closely-monitored clinical trials. However, trial-derived *efficacy* may exceed observed “real-world” *effectiveness* (62). Thus, as clinics provide decriminalized psychedelic-assisted therapies in Oregon in early 2023, analyses that address real-world use will be needed. Health economists can work with health services researchers to integrate information on costs with assessment of clinical outcomes for operating programs.

### Cost-benefit analysis

(CBA) is another powerful tool for estimating “value for money.” By contrast with CEA, in CBA, both health and non-health outcomes are valued in monetary terms. The result of CBA is expressed as a net benefit (benefit minus costs), as a benefit-cost ratio or internal rate of return. CBAs have at least two advantages over CEAs. First, by expressing outcomes in dollars, CBAs come closer to reflecting a societal welfare function (63). CBA thus makes it easier for policy makers to identify investments that have the highest societal returns, and to allocate limited budgets accordingly. Secondly, by eliminating recourse to abstract measures of outcomes such as QALYs, CBAs express results in intuitive language, such as, “For every dollar spent on X the payer will save Y dollars.” The choice between CEA or CBA for any given analysis depends on the policy question. If considering the incremental value of a psychedelic vs. a conventional treatment, CEA will suffice. However, if the question pertains to a broader set of options, including a range of outcomes beyond health, CBA is more flexible and robust.

### Scale-up and budget impact analysis

CEAs and CBAs do not quantify the *overall impact* of an intervention on health care budgets, or on the health of populations such as Medicaid beneficiaries or the members of a private health plan. Scale-up and budget impact analyses provide information that insurers and other decision makers need prior to adopting a new therapy. By outlining the nationwide public health and economic impact, they can also help make the case to NIH to fund high-quality research, and to state legislatures and health departments to facilitate access to newly legal therapies.

These models portray the likely trajectory over time of increased access to treatment, the cost of serving those patients, potential net savings in reduced medical care costs, and associated health care benefits such as QALYS gained or deaths averted. In addition, budget impact models can include payer-specific metrics such as percent of annual expenditures represented by the new intervention.

A challenge in developing comprehensive scale-up models is properly portraying supply and demand constraints. On the supply side, a key issue is the rate at which therapists can be certified. As of January 2021, 13 training programs existed across the U.S. The MDMA training program offered by the Multidisciplinary Association for Psychedelic Studies (MAPS), a leading non-profit in the field, had trained or enrolled 1,800 therapists as of November 2021. Scale-up models should include updated estimates of the number of therapists that can be certified within a regulatory and certification environment which is itself rapidly evolving. Scale-up models also need to estimate the percentage of therapists' practice that they are willing to devote to psychedelic therapy. According to a 2021 survey, 75 percent of therapists reported they would be unlikely to provide psychedelic therapy if it meant a reduction in income (64). A modeling effort by the Boston Consulting Group estimated that 22,000–40,000 MDMA-certified therapists would be needed to treat 400,000 PTSD patients by 2031 (64). Considering psychedelic medicine more broadly, this implies that treating one million patients per year by 2031 would require 55,000–100,000 newly-trained therapists in 10 years, approximately 10–17% of the US mental health workforce (64). The rate at which practitioners can be trained and effectively deployed is also a function of the prevalence of skepticism and thus reluctance to participate. This skepticism remains substantial among key clinician groups such as psychiatrists (65) and psychologists (66).

Estimates of effective demand are similarly uncertain. No previously illegal psychedelics have yet been approved as medicines by the FDA or other regulatory agencies. It is unknown which compounds will be approved on what timeline and for which specific psychiatric indications. Although recent surveys suggest a positive attitude toward psychedelic therapy and research among a majority of Americans affected by mental health issues, especially among the young, (67, 68), little is known about the percent of patients who would be willing to undergo a therapy that entails dramatic alterations in perception, and the possibility of confronting painful emotional content. Residual stigma and cultural associations with psychedelics may also discourage people from seeking treatment. On the other hand, it is reasonable to suppose that if treatments are successful, and thus become increasingly endorsed by mainstream institutions and *via* word-of-mouth, many who were originally reticent will avail themselves of these therapies.

In view of these uncertainties, initial models will need to portray ranges and be updated as the interacting dimensions of clinical effectiveness and legislative, economic, and cultural contexts change over time. Despite these qualifications, models can usefully describe the upper and lower bounds of economic and public health impact for particular medicine-disorder pairs (e.g., MDMA for PTSD) given plausible scale-up scenarios (49). Analyses of the economics of esketamine, an FDA-approved medicine with psychedelic properties, for the treatment of depression, can also inform many aspects of the economic assessment of other psychedelic therapies including scale-up and budget impact models (69).

### Price evaluation

Providers of psychedelic therapies including therapist groups and larger provider networks, as well as insurers, must understand the supply, demand, and price dynamics of these interventions. Appropriate pricing is crucial for patient access, payer adoption, and revenue generation. There are various methods for setting the price of new pharmaceuticals. Value-based approaches seek to develop a societal value estimate as an upper-bound for the price; return on investment (ROI) approaches determine the lower bound. The societal value, in turn, depends on appropriate cost-effectiveness analyses that compare net health care costs with expected health benefits (50, 70). Within plausible price bounds, the profit or revenue-maximizing price is determined by a variety of factors including especially the price elasticity of demand (71). The successful introduction of psychedelic therapies is similar to the rollout of conventional medical therapies. However, an important difference arises from the history of prohibition: Illegal cannabis continues to compete with legal, regulated cannabis products. Similarly, market models for legal psychedelic medicines must account for potential downward pressures on price exerted by well-entrenched informal markets for LSD, MDMA, psilocybin mushrooms, and other psychedelic materials.

With the implementation of Oregon's Propositions 109 and 110 in 2023, psilocybin services will become available to people who are not seeking psychiatric treatment but rather, seek support for other purposes such as personal growth or spiritual development (72). These novel services combine provision of newly-decriminalized and powerful psychoactive materials in a supportive context which is neither traditional psychotherapy nor the mere monitored provision of psychiatric medicines such as SSRIs. Provision of these services might require a lower level of professional certification, and third party payers are unlikely to reimburse for these non-medical services. These factors suggest a different price point from that of potentially reimbursable clinical provision of psychedelic-assisted psychotherapy. There will be a demand for financial analysis to help establish their cost structure and a viable price in a rapidly evolving competitive environment.

Organizations which have adopted public-benefit models for the sale of psychedelics for therapy must balance two competing goals: All else equal, lower prices mean greater access to treatment and greater public health benefit. However, lower prices also mean less revenue to direct back to non-profit research and educational activities. Many of the main actors are concerned with identifying the welfare maximizing price, not the profit-maximizing price. This is a calculation with greater uncertainties.

### Methods development

Current tools of health economic evaluation cannot assess certain issues that arise for psychedelic-assisted therapies. For example, the traditional concept of health state “utility,” roughly equivalent to “satisfaction” (73) may underestimate the benefits of psychedelic therapies. Utility ranges from, 0.0 signifying death, to a maximum of 1.0, which signifies the absence of disease. Utility is thus not equipped to reflect sustained, enhanced access to such positive experiences as awe, compassion, self-efficacy, and affinity with nature. These states, which may persist long beyond the acute effects, are reported as a result of ingestion of psychedelic materials in both clinical and naturalistic settings (74–76). Because positive cognitive states are not restricted to exposure to psychedelics, this innovation has implications for health economic evaluation generally. Capitalizing on the work on determinants and measurement of happiness and other positive states that has been developing over the past 20 years (77, 78), it would move the field away from traditional measures of health-state utility and into alignment with broader measures of welfare (79, 80). The methodological problem of developing a validated measure of overall well-being that integrates health-state utility with other measures of well-being that include positive emotional and cognitive states, has not been solved. As a first step, data should be collected from multiple sites on both “utility” and positive states so that the relationship between them can be quantified. Success would be aided by cross-disciplinary collaboration between health economist's psychologists, happiness researchers, and psychometric experts.

A second issue is standardization and comparability. To ensure both comprehensive analyses and comparability of results, health economists might establish and promulgate best practice guidelines for the conduct of economic analyses of psychedelic therapies. These might focus on the implementation of a subset of the recommendations of the Second Panel and costing guidance from the Global Health Cost Consortium (81, 82). As mentioned above, among these are methods to estimate broad societal benefits such as increased well-being of clients' family members; and important secondary effects such as reductions in domestic violence, substance use disorder; and involvement with the criminal justice system.

Third, unlike standard psychiatric therapies many of which are continuous, economic models for psychedelic therapies need to reflect the incremental costs and benefits of irregular episodic treatment. As long-term outcome data become available, it will be important to construct models that portray changing probabilities of treatment success following relapse.

The increased acceptance of psychedelics for medical use may have externalities, both negative and positive. Recent surveys show a marked increase in the use of psychedelics in the United States. According to the National Survey on Drug Use and Health (NSDUH), between 2015 and 2019 there was an increase in hallucinogen use from 4.69 million to 6.01 million, including a 60% increase in the use of LSD and a 96% increase in the use of other hallucinogens (83). Thus, a fourth area requiring innovative measurement and modeling approaches is quantifying the public health and cost impacts of increased access to psychedelic materials outside of clinical settings.

We are in an era of unprecedented tolerance and perceived legitimacy of psychedelics. This climate of favorable opinion is conferred by reports from FDA-sanctioned clinical trials, the establishment of academic research centers at prestigious institutions, the influx of private investment, decriminalization in some jurisdictions, and the return of NIH funding for psychedelic research. In this environment, it is reasonable to assume that an increasing number of new users will consume psychedelic materials for personal development, celebratory and spiritual purposes, and unadorned recreation. Working with epidemiologists and research methods experts, health economists can help interpret the burgeoning literature on the mental and physical health effects on these new users. Among the key questions: Given the quality of extant research including cross-sectional designs, reliance on self-report and other potential sources of bias in many studies, what does the evidence as a whole suggest about access to psychedelics as an independent cause of positive or negative health effects? Is it possible to model the effects of psychedelic use in naturalistic settings on health care costs and outcomes?

## Principles to guide the health economics research program for psychedelics

We believe that the potentially transformative effects of psychedelics in mental health treatment warrant a proactive economics research agenda. We propose the following characteristics.

### Forward-looking / anticipatory

Cost-effectiveness analyses are often considered only after promising results from clinical trials. There is logic to this: Why devote resources to cost-effectiveness analysis when effectiveness has not yet been established? However, more time than necessary thus elapses between promising clinical findings and the publication of associated economic analyses. The consequence is that the adoption of new therapies and the benefits they confer may be delayed. We advocate a middle ground between premature economic analysis and delaying work until definitive clinical results are available. In addition to more rapid dissemination of important economic findings, by establishing early collaboration with clinical researchers, the quality of the economic analysis stands to benefit since appropriate economic data collection instruments and methods can sometimes be woven into the design of the research. As the clinical research develops over the next several years greater knowledge will be gained on a number of factors that affect both clinical and economic outcomes. For example, as information is gained over time on the long-term durability of benefits and the potential effects of multiple treatment sessions for those who do not respond to the initial regimen, the associated economic analyses will need revision. Thus, the economic analysis of a particular therapy will rarely be final and definitive. Rather, economic assessments will evolve to reflect the increasing refinement of the clinical knowledge.

### Equity

Within the field of economics research on psychedelic therapies, the same efforts to achieve broad representation that are applied to other academic fields are also pertinent. Furthermore, economic research on psychedelics should regularly consider equity issues such as need for mental health treatment, access to psychedelic interventions, and differential clinical and economic effects by economic status. In order to avoid an implementation outcome in which those most in need have the least access, health economists should include analyses of how the realities of health care financing in the United States affect access to psychedelic therapies. Working with health services researchers to devise reimbursement plans that guarantee equitable access should be high on their agenda. For example, equitable access will require that large private and public payers including, in the U.S., Medicaid, reimburse therapists adequately for psychedelic-assisted therapy sessions. Much depends on Current Procedural Terminology codes and other insurance billing codes that are designated for the new therapies, and the reimbursement associated with those codes. If too low, practitioners will have insufficient incentive to participate. In that case, while formally an insured benefit; psychedelic therapies will remain unavailable to most of the population who could benefit. Health economist has a role to play in generating realistic estimates of the supply of accessible psychedelic therapy available across a range of reimbursement levels.

### Global scope

To date, clinical trials of psychedelic-assisted therapies have been conducted in the USA, Europe, Australia, New Zealand and Israel, yet 84.3% of people affected by mental illness live in low and middle-income countries (84, 85). On the effectiveness side, it will be important to understand if the benefits reported in rich-countries are replicated in different cultural contexts. Some lower-income countries have traditional practices using plant-based psychedelics that long pre-date use in the West. It is not clear whether western-style psychotherapeutic modalities will be appropriate or effective in these contexts. Models developed for rich countries may need to be revised or re-thought entirely for application in low- and middle-income income countries. Delivering psychedelic therapies will cost less in less wealthy countries. However, the potential savings in future medical care costs will also be lower, leaving an unknown effect on net discounted costs.

### Teaching, mentoring, and partnerships

To ensure the emergence of a cadre of researchers prepared to further advance this agenda, health economists should help to develop courses on economics and implementation science for psychedelic therapies. Through partnerships with leading individual researchers and institutions overseas, they can also develop an appropriate psychedelics-related economics research agenda in other countries, including middle and low-income countries.

### Scientific rigor

The last few years have seen a rapid rise of interest in psychedelic-based therapies by the private sector and a concomitant influx of research dollars. Venture capital investments for 2020 and 2021 combined was $31.2 million compared with $49.5 million in the previous 5 years (86, 87). These expenditures eclipse the budgets of the non-profit entities that dominated the early period of the new era of psychedelic science. MAPS, for example, spent $18.6 million in FY 2020 (88). In the decriminalized setting of Oregon, likely to be followed soon in other states, it is easy to imagine how marketing hype could supplant evidence-based practice (89). Thus, patients, practitioners, researchers and health care payers need a countervailing body of objective health services research and economic analysis with a minimum of real or perceived conflicts of interest, and a commitment to Open Science (90). For these reasons, despite the influx of private research investments, there will be an ongoing role for NIH, other government funding, and philanthropic assistance for arms-length support to leading researchers.

## Conclusion

Encouraging results from clinical trials of psychedelic therapies for major mental health disorders suggest that psychiatry may soon expand the range of effective treatments. Findings on effectiveness are now sufficiently advanced that research on the economics of these emerging therapies is timely and needed. Among priority areas for economic analyses are cost-effectiveness and cost-benefit analyses that assess value for money to payers and society at large; scale-up models that portray the cumulative impact of access to psychedelic therapies; and price and market analyses that health care providers and payers need to plan the delivery of care.

## Author contributions

EM: conceptualization, methodology, investigation, formal analysis, project administration, supervision, visualization, writing—original drafts, and writing final draft. JK: conceptualization, methodology, formal analysis, validation, visualization, writing—original drafts, and writing final draft. SB: conceptualization, formal analysis, and writing final draft. All authors contributed to the article and approved the submitted version.

## Conflict of interest

The authors declare that the research was conducted in the absence of any commercial or financial relationships that could be construed as a potential conflict of interest.

## Publisher's note

All claims expressed in this article are solely those of the authors and do not necessarily represent those of their affiliated organizations, or those of the publisher, the editors and the reviewers. Any product that may be evaluated in this article, or claim that may be made by its manufacturer, is not guaranteed or endorsed by the publisher.

## Footnotes

^1^Disability-Adjusted Life-Years (DALYs) is a similar metric. QALYs are usually applied in developed world settings; and DALYs are applied in developing country settings.
